# Geriatric scores can predict long-term survival rate after hip fracture surgery

**DOI:** 10.1186/s12877-019-1223-y

**Published:** 2019-08-01

**Authors:** Carmen da Casa, Carmen Pablos-Hernández, Alfonso González-Ramírez, José Miguel Julián-Enriquez, Juan F. Blanco

**Affiliations:** 1 Instituto de investigación biomédica de Salamanca (IBSAL), Salamanca, Spain; 2 Othogeriatric Unit, Hospital Universitario de Salamanca, Salamanca, Spain; 3 Trauma and Orthopedic Surgery Department, Hospital Universitario de Salamanca, Paseo San Vicente, 58-182, 37007 Salamanca, Spain

**Keywords:** Comprehensive geriatric assessment, Cumulative survival rate (CSR), Readmission, Katz index, Lawton-Brody index

## Abstract

**Background:**

The management of hip fractures is nowadays mainly performed in Orthogeriatric Units, one of whose fundamental tools is the application of geriatric scores. The purpose of this study is to establish the potential usefulness of Barthel Index, Katz Index, Lawton-Brody Index and Physical Red Cross Scale geriatric scores as predictors of survival rate and readmission rate in older patients after hip fracture surgery.

**Methods:**

We designed a prospective single-center observational study, including 207 older adults over age 65 who underwent hip fracture surgery in the first half of 2014 and followed up to September 2018. Cumulative survival and readmission rates were analyzed by Kaplan-Meier; group comparison, by Log-Rank and hazard ratio, by Cox regression.

**Results:**

We found statistical differences (*p* < 0.001) for cumulative survival rate by every geriatric score analyzed (BI HR = 0.98 [0.97,0.99]; KI HR = 1.24 [1.13–1.37]; LBI HR = 1.25 [1.16, 1.36]; PCRS HR = 1.67 [1.37,2.04]). Furthermore, we could determinate an inflection point for survival estimation by Barthel Index (BI 0–55/60–100*, *p* < 0.001, HR = 2.37 [1.59,3.53]), Katz Index (KI A-B*/C-G, p < 0.001, HR = 2.66 [1.80, 3.93], and Lawton-Brody Index (LBI 0–3/4–8*, p < 0.001, HR = 3.40 [2.09,5.25]). We reveal a correlation of the Charlson Index (*p* = 0.002) and Katz Index (*p* = 0.041) with number of readmissions for the study period.

**Conclusions:**

The geriatric scores analyzed are related to the cumulative survival rate after hip fracture surgery for more than 4 years, independently of other clinical and demographic factors. Katz Index in combination with Charlson Index could also be a potential predictor of the number of readmissions after surgery for hip fracture patients.

**Electronic supplementary material:**

The online version of this article (10.1186/s12877-019-1223-y) contains supplementary material, which is available to authorized users.

## Background

Hip fracture in older people remains one of the most important health problems. The high incidence, morbidity and mortality associated with hip fractures have made it necessary to establish specific strategies for the prevention and management of its injury. Currently, the management of older adults with hip fracture is mainly performed in specific units called “Orthogeriatric Units” that can improve the outcomes of this group of patients [1, 2]. One of the fundamental tools for the management of patients in these units is the so-called Comprehensive Geriatric Assessment (CGA), that collects data on various clinical and functional aspects of patients through the application of different scores [3, 4]. Barthel Index (BI), Katz Index (KI) and Lawton-Brody Index (LBI) scores are broadly used in a standardized way as part of the CGA and for the evaluation of mobility, CGA could also include a widely disseminated score in Spain, the Physical Red Cross Scale (PCRS). These geriatric scores allow us to know the functional situation of the patient, what, taken together with clinical data, allow us to establish the clinical-functional situation of each patient and propose appropriate treatment.

For the evaluation of the prognosis of older patients with hip fracture, some tools have shown predictive capacity. Hence, NHFS, O-Possum, and other indexes have been developed and are in use [5]. Among the various risk factors associated with higher mortality in older patients with hip fracture are biodemographic factors, such as male gender and age, clinical factors such as ASA class for anesthetic risk or comorbidity rates, and care factors such as surgical delay [6, 7], but also previous functional situation could have been related with short-term mortality [8, 9] and some geriatric scores have previously studied as predictors of short-term mortality for patients undergoing surgery [10, 11].

The purpose of this study is to establish the possible usefulness of the geriatric scores BI, KI, LBI and PCRS as predictors of long-term survival rate in older patients with hip fracture. We also aim the detection of a possible point from which the survival outcome is compromised by the most significant way.

## Methods

### Design and population

We designed a prospective observational study, including all patients 65-years-old and older with hip fracture admitted in 2014 from January 1st to June 30th in the orthogeriatric unit of the University Hospital of Salamanca (UHS). We excluded those cases that were not treated surgically, as well as all those patients who suffered fractures due to high energy trauma or who showed tumoral etiology. Patients who could not move even with technical aid before the fracture, corresponding with 5 score on the PCRS were also excluded. All patients included in the study expressed their consent to participate.

Overall, it is a cohort of 207 patients undergoing surgery after hip fracture in the first half of 2014, admitted to the Trauma and Orthopedic Surgery Service of the UHS.

### Demographic and clinical variables

On admission, sociodemographic variables such as gender, date of birth and admission, and place of residence were collected. We also recorded the type of fracture, as well as the ASA preoperative anesthetic classification for each patient, and comorbidity data were collected determining a score according to Charlson Index (CI). During the hospitalization, the type of procedure and destination at discharge were also recorded.

For the study of long-term survival, a case was defined by each patient who died and follow-up was determined until last hospital contact before September 2018, with a maximum follow-up of 55 months. Likewise, re-admissions were recorded in our hospital until September 2018. A re-admission was recorded once the patient was in-hospitalized after discharge. Time until first re-admission was counting from the first discharge date.

### Geriatric scores

In the UHS Orthogeriatric Unit, the following geriatric scores are used as part of CGA: Barthel Index, Katz Index, Lawton-Brody Index and Physical Red Cross Scale.

The BI, described by Mahoney and Barthel in 1965 [12], collects data on the degree of capability for the development of 10 basic activities of daily living (ADL). For each activity analyzed, a gradual score is applied in 5 points, according to the patient ability to perform it, stratifying the patients into five categories: total dependence (< 20 points), severe dependence (between 20 and 35 points), moderate dependence (between 40 and 55 points), slight dependence (between 60 and 95 points) or complete independence (100 points).

The KI also performed in the 1960s by S. Katz [13], was designed for the evaluation of patients with hip fracture. It estimates the dependence or independence of the patient to perform basic ADL in a similar way to the IB. Specifically, it analyses 6 functions from which categorization of patients originates, possible situations ranging from the total independence named with the letter A to the total dependence indicated with the letter G.

A few years later, Lawton and Brody developed an index for evaluating instrumental activities of daily living (IADL) [14]. The LBI assigns a score from 0 to 8; the highest score indicates the better functional capability. Due to the characteristics of the IADL analyzed, LBI has been traditionally applied differentiating the gender of the patient and limiting to 4 the maximum score for men [15]. At UHS, the whole questionnaire was determined regardless of patient gender.

The PCRS score evaluates the physical ambulatory ability for the patient. It was developed at the Red Cross Hospital in Madrid [16] on the ‘70s and nowadays it uses is declining due to the implementation of other non-Spanish scores as the Functional Ambulation Classification (FAC). The original Red Cross Scale has a mental-status evaluation, not analyzed in our center, and a physical-status evaluation, concerning 5 levels of ambulatory ability from 0, which indicates full capability, to 5, which indicates any ambulatory capability. This score was also used as exclusion variable in our study for those patients with no ambulatory ability (PCRS = 5).

### Statistical analysis

Data were imported into a database for statistical analysis with the IBM SPSS Statics program (v.23). Descriptive statistics included mean, standard deviation and range, and normality of sample distribution was defined by the Kolmogorov-Smirnoff test. We ascertain the statistically significant differences among groups by non-parametrical test.

The cumulative survival analysis was performed by the Kaplan-Meier test. The same test was used to study time until first re-admission. Group comparison by factor was estimated using the Log-Rank test, and hazard ratio estimation (HR) was performed by Cox regression analysis, indicating a 95% confidence interval. The reference category was always selected for the best functional status.

For the determination of the inflection point in the survival trend, we made all possible groupings and studied them by linear regression analysis. The inflection point was defined by the greatest mean difference in those linear regression analyses of the pooled sample. Validation of the inflection points consisted of the checkup of the inflection points on the restricted population regarding its significance.

In all cases, a *p*-value less than 0.05 was considered statistically significant.

## Results

The study population was composed of 207 patients. Most cases were women (82.1%) and the mean age was greater than 85 years old (Table 1).

**Table 1:** Biodemographic and clinical features of patients

Biodemographic features		Clinical variables	
Gender		Charlson Index (CI)	
Female	82.1%	0–1 (no comorbidity)	54.1%
Male	17.9%	2 (med. comorbidity)	16.9%
Age		≥3 (high comorbidity)	29.0%
65–79 years-old	17.9%	ASA class	
80–89 years-old	49.8%	I-II	36.7%
> 90 years-old	32.4%	III-IV	63.3%
Residence		Type of fracture	
Town	51.2%	Intracapsular	36.7%
Rural	48.8%	Trochanteric	49.8%
Institution-living		Periprosthetic	1.4%
At admission	33.8%	Other	12.1%
At discharge	50.7%	Surgical procedure	
Side of fracture		Osteosynthesis	62.3%
Right	44.4%	Partial hip replacement	36.7%
Left	55.6%	Total hip replacement	1.0%

Descriptive analysis showing frequencies of studied variables, on the 207 patient study cohort. Med: medium

We also analyzed the place of residence of patients, and in Table 1 is shown that 51.2% of patients were residents from municipalities with more than 12,500 inhabitants and 33.8% of participants were living on an older people specific healthcare institution. 16.9% of non-institutionalized patients at admission were institutionalized after discharge. Regarding the comorbidity of patients, it was evaluated by Charlson Index (CI) and we described three groups of patients concerning no comorbidity or slight comorbidity (CI = 0/1), medium comorbidity (CI = 2) or high comorbidity (CI = 3), beside the ASA preoperative anesthesia risk assessed (Table 1).

Incidence by type of hip fracture was also studied, showing that most cases it was a trochanteric fracture, whose surgical intervention treatment was osteosynthesis-based. 55.6% of surgical procedures were for the left side (Table 1).

Afterward the interview of patients and their accompanying persons by the geriatric team, the descriptive data of the CGA performed is displayed on the first column of Additional file 1: Table S1. Descriptive data by gender of LBI is also shown in Additional file 1: Table S2.

### Part 1. Survival

We analyzed cumulate survival for patients included in this study until last hospital contact before September 2018. That way, we avoid the bias for non-recorded mortality in the course of more than four years covered by our study.

**Fig. 1:**
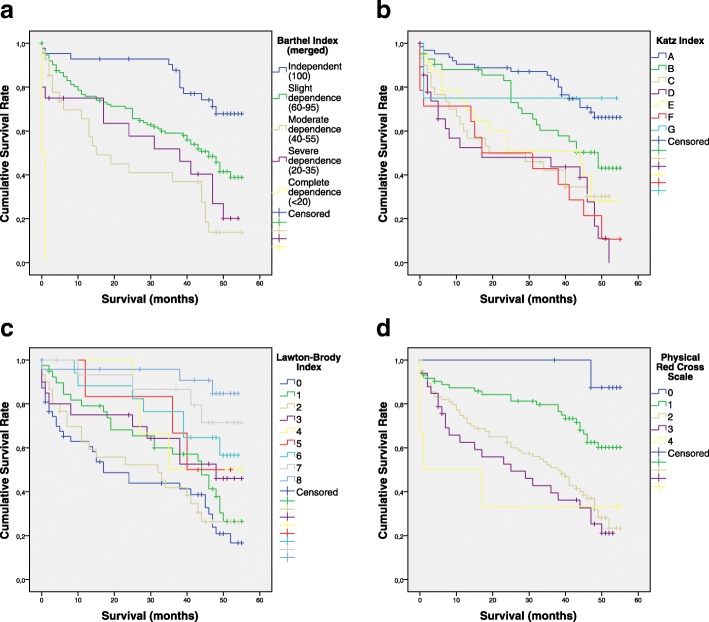
Survival outcome after hip fracture surgery. From left up, cumulative survival representation for 55 months after hip fracture surgery on Barthel index merged categories (*p* < 0.001), Katz index categories (*p* < 0.001), Lawton-Brody index score (*p* < 0.001) down left, and Physical Red Cross Scale score (*p* < 0.001)

We found statistical differences (*p* < 0.001) for every geriatric score detailed in the CGA with cumulative survival rate (Fig. 1). Additional file 1: Table S1 shows estimated survival for each category reviewed by Kaplan-Meier method.

Finding out more, we tried to determinate the Hazard Ratio (HR) associated with those dependence evaluations studied by the geriatric scores. We observed that in BI, besides revealing differences among groups, there was no great hazard (HR = 0.98 [0.97,0.99], reference category: “independent”). Conversely, analyzing KI (HR = 1.24 [1.13–1.37]), patients in A category (reference category) who have total independence for ADL, showed a lower risk. Similar results were observed for LBI (HR = 1.25 [1.16, 1.36], reference category: 8), and PCRS (HR = 1.67 [1.37,2.04], reference category: 0).

### Part 2. Inflection point in survival rates

Reviewing previous results on survival rates by Kaplan-Meier test, we reasoned the possible appearance of an inflection point on the geriatric scores from which the survival rate of patients after hip fracture surgery could be significantly abridged. It would be interesting to know if there is a functional disability discrete score from which survival was reduced.

**Fig. 2:**
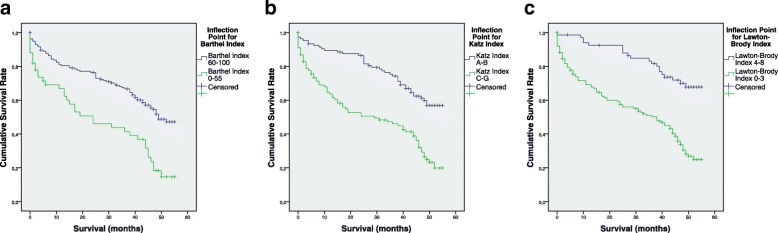
Geriatric scores inflection points survival outcomes. From left, cumulative survival representation for 55 months after hip fracture surgery on Barthel index inflection point (0–55 vs. 60–100) *p* < 0.001, Katz index inflection point (A-B vs. C-G) *p* < 0.001 and Lawton-Brody index inflection point (0–3 vs. 4–8) *p* < 0.001

Evaluating survival distribution for patients on different categories of BI, we found the greatest difference of mean survival rate between patient group presenting moderate and severe dependence (BI 0–55) and patients showing slight dependence or total independence (BI 60–100). Hence, Fig. 2 shows survival outcome for those groups (*p* < 0.001), whose HR was 2.37 [95%CI = 1.59,3.53], as reference category was BI 60–100.

Regarding KI, we could establish an inflection point statistically significant (p < 0.001) between patients displaying great independence (KI A-B) and the other scored patients. Figure 2 shows respective survival outcomes, whose associated HR was 2.66 [1.80, 3.93] (reference category KI A-B).

According to LBI, Fig. 2 also shows survival outcomes for patients scored 3 or less and patients scored 4 or more (*p* < 0.001). The analysis including the whole population studied revealed a 3.40 HR [2.09,5.25], but also detailed exploration also reveal a greater HR in men population (HR = 5.46 [1.79,16.68], *p* = 0.003) and a significant HR in women population (HR = 3.11 [1.81,5.36], *p* < 0.001). Category reference in all cases was LBI 4–8.

Concerning ambulatory physical capability measured by PCRS, even though statistically significant differences were observed on mean survival rates among the different categories studied, due to the limited extreme values obtained, it is tough to certainly determinate an inflection point. Consequently, we could not establish an inflection point for PCRS on our studied population.

### Part 3. Inflection point validation

According to the previous results, we aimed the validation of the inflection points determined on survival rates by geriatric scores from CGA above and beyond the clinical and demographic factors recorded in the study. Table 2 shows a check validation summary assessing the *p*-values on Log-Rank test.

**Table 2:**
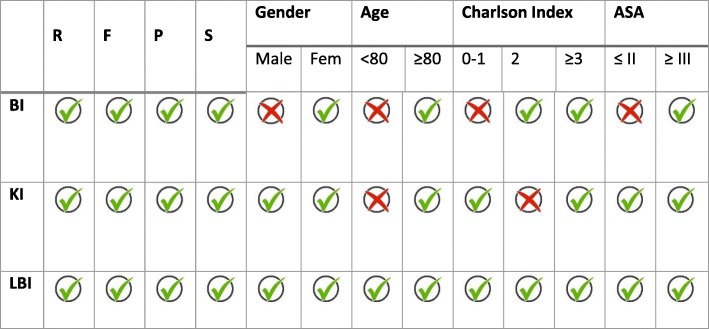
Validation of geriatric scores inflection points

BI: Barthel index inflection point (0–55/60–100); KI: Katz index inflection point (A-B/C-G); LBI: Lawton-Brody index inflection point (0–3/4–8). R: residence; F: type of fracture; P: surgical procedure; S: side of fracture; Fem: female. A positive check indicates *p*-value <0.05 on Log-Rank test for each category

The institution-living status at admission was also analyzed as a confounding variable for the inflection point validation. We cannot assume that the inflection points described before could be used to assess the long-term survival of institutionalized patients (*p* > 0.05).

Nevertheless, valuations of the change in the survival trend grouped in the different geriatric scores validate LBI inflection point (0–3/4–8) in all fields studied: KI inflection point (A-B/C-G), on population over 80 years-old with a slight or high comorbidity (IC ≠ 2); and BI inflection point (0–55/60–100) in women older than 80 years-old with medium or high comorbidity, or an elevated ASA grade (III / IV).

For more detailed information, see Additional file 1: Table S3, showing adjusted HR for all categories studied on Cox Regression analyses.

### Part 4. Readmission rates

As re-admission of patients at University Hospital of Salamanca data was recorded, we could analyze 195 cases (12 patients drop out of the study). A great percentage of patients (44.4%) did not require any admission along the four years covered in our study, nevertheless 9.7% of patients were re-admitted on the first 30 days after discharge (not only in the orthogeriatric unit but also in other hospital services) and 13.8% of patients were re-admitted on the first 3 months after hip fracture surgery.

According to the number of readmissions on these four years, we tried to determinate a correlation with the functional status previous to hip fracture of patients. Nonetheless, we could not evince a significant correlation between the number of readmissions and the different geriatric scores analyzed (BI-*p* = 0.102; KI-*p* = 0.139; LBI = 0.739; PCRS-*p* = 0.803), but we reveal a statistically significant correlation between the CI score (not merged) and readmissions during the following 4 years after hip fracture surgery (ϐ = 0.197; R^2^ = 0.039; *p* = 0.006). Remarkably, there is another regression modeling for CI (*p* = 0.002) in combination with KI (*p* = 0.041) that could lightly improve the previous result (R^2^ = 0.059).

Reviewing time until the first readmission, the meantime until the first readmission of patients in our study was 7.61 (±13.13) months. Once more, we could not reveal significant differences among that item and most of the geriatric scores analyzed (BI – *p* = 0.066; LBI – *p* = 0.438; PCRS – *p* = 0.511), but also there was a significant difference on KI categories (*p* = 0.033) and time until first readmission (Additional file 1: Figure S1). However, we could not establish a significant correlation for those differences (*p* = 0.766).

In addition, we tried to correlate the inflection points established for survival trend with time until the first readmission, but we could not evince any significant result (*p* > 0.05).

## Discussion

Our study allows firstly to propose the use of these geriatric scores as predictors of long-term survival after hip fracture surgery, being able to establish inflection points that allow us to obtain more detailed knowledge about the prognosis of these patients in terms of survival.

Hip fracture in the older patient is still a very relevant health problem. Increase of the older population became it a high incidence health problem [17, 18]. In recent decades, significant improvements have been established in the management of hip fracture in older patients. The importance of early surgery or the establishment of effective collaboration between geriatrics and traumatology with the implementation of the orthogeriatric units have improved the outcomes [1, 2]. One of the advantages of this collaboration is the CGA that allows us to better understand the functional and clinical situation of each patient, thus being able to apply the appropriate therapeutic measures to each case [3, 19]. The CGA uses the so-called geriatric scores that can offer an overview of the patient functional status. The usefulness of these scores is widely documented in the scientific literature [20, 21] and, in a general way, they offer information on the degree of dependence or independence of patients for carrying out some activities.

As occurs when managing other health problems, results’ prediction, more specifically mortality or survival, is a relevant topic. Several studies analyze various risk factors of mortality in older patients with hip fracture [22, 23] and also showing that functional situation prior to admission could be related to prognosis [24, 25].

Our study shows, as other authors pointed out, how functional status can affect long-term survival. It is noteworthy how the degree of mobility prior to fracture is an important prognostic factor.

Of the four scores studied, the Katz index (KI) seems to offer the most relevant information. KI indicates the independence of the patient for carrying out basic ADL, it is, therefore, a reflection of the basic functional situation of the patient. It seems logical therefore that it could be related to the prognosis, indeed some works have shown that the assessment of ability to perform ADL is related to the short-term mortality [26]. In this sense, our study provides information on the fact that survival increases in the A-B group of the KI, in other words, in those patients whose functional status is the best. When patients are under 80 years of age, the inflection point loses significance and we should only take into account the estimated overall survival and not the inflection point. Similarly, when analyzing BI, we can differentiate independent patients presenting better prognosis from other patients. In this case, age and degree of comorbidity also influence the inflection point.

LBI introduces a bias in our country since the instrumental ADL that it analyses were carried out traditionally mainly by women. Although the study was also conducted segregating gender of the patient, this factor must be taken into account in order to reveal its potential survival prediction power. Finally, the PCRS seems to be a predictor of survival with statistical significance. This aspect, previous mobility before hip fracture, has also been analyzed by other authors using diverse tools [27]. Ambulatory ability somehow informs about independence grade of patients and also comes to complete what seemed obvious: the better functional status, the better prognosis.

In addition, we exhibit a readmission rates study, showing limitations of the appliance of those geriatric scores. Again, KI score became relevant to predict how long could it take until the first readmission after hip fracture surgery discharge. We also reveal that CI score for comorbidity could be taken alone or with KI to assess readmission rates, what is an interesting new application for that recognized indexes, as previously pointed Härstedt et al. [28].

Our study presents some limitations, mainly the size of the population studied and its common provenance. On the contrary, patient follow-up is prolonged and allows us to compare several assessment tools commonly used in the CGA as predictors of survival and readmission.

## Conclusions

The geriatric scores analyzed (BI, KI, LBI and PCRS) are related in a statistically significant way to the long-term survival rate after hip fracture surgery, independently of other clinical and demographic factors. In addition, for the scores analyzing ADL and IADL, inflection points in the survival trend are defined.

KI score is also statistically significant related to time until the first readmission for patient suffering hip fracture surgery. CI, and combination of CI and KI can also be a potential predictor of the number of readmissions after surgery for hip fracture patients.

Although the relationship among geriatric scores and hip fracture patient’s outcomes has been shown by many authors, if we consider the long-term follow-up and inflection points defined in trend for survival rate, our results are original and could be helpful in order to establish proper therapeutic approaches, so BI, KI, LBI and PCRS could be used as survival estimators.

## Additional file

Additional file 1: Geriatric scores can predict long-term survival rate after hip fracture surgery: Supplementary data. **Table S1.** Descriptive analysis for geriatric scores studied. Survival outcomes and estimation. **Table S2.** Lawton-Brody Index frequencies by gender of patient. **Table S3.** Inflection points validation. Adjusted HRs. **Figure S1.** Time until first readmission (months). Outcome on Katz index categories. (DOCX 102 kb)

## Data Availability

The data supporting this study are available from the corresponding author upon reasonable request.

## Abbreviations

ADL: Activities of daily living
BI: Barthel index
CGA: Comprehensive geriatric assessment
CI: Charlson index
FAC: Functional Ambulation Classification
HR: Hazard ratio
IADL: Instrumental activities of daily living
IP: Inflection point
KI: Katz index
LBI: Lawton-Brody index
PCRS: Physical red cross scale
UHS: University Hospital of Salamanca
