# Supplementary material for: Geriatric scores can predict long-term survival rate after hip fracture surgery
Source: BMC Geriatr. 2019 Aug 1;19:205. doi: 10.1186/s12877-019-1223-y (PMC6676564; doi:10.1186/s12877-019-1223-y)
Supplement: Supplementary file 1 — Geriatric scores can predict long-term survival rate after hip fracture surgery: Supplementary data. Table S1. Descriptive analysis for geriatric scores studied. Survival outcomes and estimation. Table S2. Lawton-Brody Index frequencies by gender of patient. Table S3. Inflection points validation. Adjusted HRs. Figure S1. Time until first readmission (months). Outcome on Katz index categories. (DOCX 102 kb) [file 12877_2019_1223_MOESM1_ESM.docx]

Additional file

# Geriatric scores can predict long-term survival rate after hip fracture surgery.

Contents

[Table 1. Descriptive analysis for geriatric scores studied. Survival outcomes and estimation. 2](#_Toc13481280)

[Table 2. Lawton-Brody Index frequencies by gender of the patient 4](#_Toc13481281)

[Table 3. Inflection points validation. Adjusted HRs. 5](#_Toc13481282)

[Figure 1. Time until first readmission outcome on Katz index categories 6](#_Toc13481283)

## Table S1. Descriptive analysis for geriatric scores studied. Survival outcomes and estimation.

|  | Frequency | Survival rate (months) | | KM estimation  (months)  [95% CI] |
| --- | --- | --- | --- | --- |
|  |  | Mean ± SD | Median[Q1, Q3] |  |
| BARTHEL INDEX | | | | |
| Independent (100) | 21.3%  (n=44) | 40.27  ±17.66 | 47.5  [37.3,52.8] | 47.7  [43.4,52.0] |
| Slight dependence (60-95) | 54.6%  (n=113) | 32.13  ±19.14 | 40.0  [11.5,49.5] | 36.0  [32.1,39.9] |
| Moderate dependence (40-55) | 13.5%  (n=28) | 21.21  ±19.48 | 13.5  [3,44] | 24.7  [16.9,32.5] |
| Severe dependence (20-35) | 9.7%  (n=20) | 26.0  ±21.33 | 27.5  [1.25,47] | 30.3  [21.0,39.7] |
| Complete dependence (<20) | 1.0%  (n=2) | 0.5  ±0.71 | 0.5  [0,1] | 0.5  [0,1.5] |
| KATZ INDEX | | | | |
| A | 31.4%  (n=65) | 39.37  ±17.11 | 47 [35.5,52] | 46.2  [42.3,50.1] |
| B | 20.3%  (n=42) | 35.64  ±17.70 | 43  [25,50.3] | 39.0  [33.4,44.6] |
| C | 14.5%  (n=30) | 24.60  ±19.39 | 18.5  [6.3,46] | 26.9  [19.4,34.4] |
| D | 13.5%  (n=28) | 20.46  ±21.27 | 7.5  [2,45.5] | 25.2  [16.5,33.9] |
| E | 11.6%  (n=24) | 27.0  ±20.20 | 24  [6,47] | 31.2  [22.7,39.4] |
| F | 6.8%  (n=14) | 25.36  ±20.85 | 24  [0.8,53] | 25.7  [14.9,36.4] |
| G | 1.9%  (n=4) | 37.0  ±24.43 | 46.5  [11.5,53] | 40.8  [18.3,63,2] |
| LAWTON-BRODY INDEX | | | | |
| 0 | 22.7%  (n=47) | 22.26  ±20.85 | 15  [2,45] | 25.8  [19.3,32.3] |
| 1 | 19.3%  (n=40) | 31.05  ±19.12 | 34.5  [12.3,48.8] | 35.3  [29.2,41.5] |
| 2 | 14.5%  (n=30) | 25.17  ±19.56 | 27.5  [5,43.25] | 27.7  [20.1,35.3] |
| 3 | 9.7%  (n=20) | 32.5  ±20.32 | 38  [12,50.75] | 36.4  [26.7,46.0] |
| 4 | 2.9%  (n=6) | 41.17  ±13.81 | 43  [26.5,54.3] | 42.0  [31.3,52.7] |
| 5 | 2.9%  (n=6) | 39.17  ±14.78 | 41.5  [30,52] | 40.7  [29.2,52.1] |
| 6 | 8.2%  (n=17) | 41.0  ±14.54 | 48  [33.5,51.5] | 43.4  [36.0,50.7] |
| 7 | 8.2%  (n=17) | 39.29  ±18.29 | 46  [31,53.5] | 48.1  [41.4,54.8] |
| 8 | 11.6%  (n=24) | 40.75  ±18.02 | 48.5  [39,52] | 50.5  [33.2,39.0] |
| PHYSICAL RED CROSS SCALE | | | | |
| 0 | 4.8%  (n=10) | 44.5  ±16.46 | 50  [44.5,53.25] | 54.0  [52.2,55.8] |
| 1 | 35.3%  (n=73) | 36.6  ±19.25 | 45.0  [25,52] | 43.6  [39.3,47.9] |
| 2 | 41.1%  (n=85) | 28.88  ±19.01 | 33  [10.5,47.5] | 32.2  [27.8,36.6] |
| 3 | 15.9%  (n=33) | 24.82  ±19.97 | 23  [5.5,47] | 27.1  [20.0,34.2] |
| 4 | 2.9%  (n=6) | 21.17  ±26.62 | 9  [0,54.25] | 21.3  [1.7,41.0] |

KM: Kaplan-Meier.

## Table S2. Lawton-Brody Index frequencies by gender of the patient.

|  | 0 | 1 | 2 | 3 | 4 | ≥4 | 5 | 6 | 7 | 8 |
| --- | --- | --- | --- | --- | --- | --- | --- | --- | --- | --- |
| Men | 24.3 | 5.4 | 18.9 | 10.8 | 8.1 | **40.6** | 5.4 | 5.4 | 8.1 | 13.5 |
| Women | 22.4 | 22.4 | 13.5 | 9.4 | 1.8 | **32.4** | 2.4 | 8.8 | 8.2 | 11.2 |

Data showing absolute percentages.

## Table S3. Inflection points validation. Unadjusted and adjusted HR on Cox Regression analyses.

|  | BI IP | KI IP | LBI IP |
| --- | --- | --- | --- |
| Unadjusted | 2.37  (<0.001) | 2.66  (<0.001) | 3.40  (<0.001) |
| Gender | | | |
| Women | 2.58  (<0.001) | 2.43  (<0.001) | 3.11  (<0.001) |
| Men | 1.704  (0.408) | 5.02  (0.001) | 5.46  (0.003) |
| Age | | | |
| 65-79 years-old | 1.94  (0.532) | 2.90  (0.082) | 3.15  (0.060) |
| ≥ 80 years-old | 2.19  (<0.001) | 2.43  (<0.001) | 3.054  (<0.001) |
| Place of residence | | | |
| Town | 2.74  (<0.001) | 2.11  (0.006) | 4.09  (<0.001) |
| Rural | 2.088  (0.012) | 3.556  (<0.001) | 2.84  (0.003) |
| Institution-living at admission | | | |
| No | 4.22  (<0.001) | 3.40  (<0.001) | 3.54  (<0.001) |
| Yes | 1.34  (0.324) | 1.55  (0.194) | 22.05  (0.306) |
| Charlson Index (CI) | | | |
| No comorbidity (CI=0-1) | 1.50  (0.255) | 2.37  (0.004) | 2.91  (0.002) |
| Medium comorbidity (CI=2) | 4.59  (0.002) | 1.67  (0.262) | 4.26  (0.054) |
| High comorbidity (CI≥3) | 2.20  (0.012) | 2.94  (0.002) | 2.81  (0.019) |
| ASA Class | | | |
| I-II | 1.77  (0.206) | 2.63  (0.005) | 2.05  (0.050) |
| III-IV | 2.35  (<0.001) | 2.52  (<0.001) | 4.65  (<0.001) |
| Surgical intervention | | | |
| Osteosynthesis | 2.54  (0.001) | 3.02  (<0.001) | 3.68  (<0.001) |
| Partial hip replacement | 2.35  (0.007) | 3.30  (0.013) | 2.87  (0.008) |

*Data showing stimated HR and p-value in brackets on Cox regression analyses. BI IP: Barthel Index inflection point (0-55/60-100*); KI IP: Katz Index inflection point (A-B*/C-G); LBI IP: Lawton-Brody Index inflection point (0-3/4-8*). Reference category marked by *.*


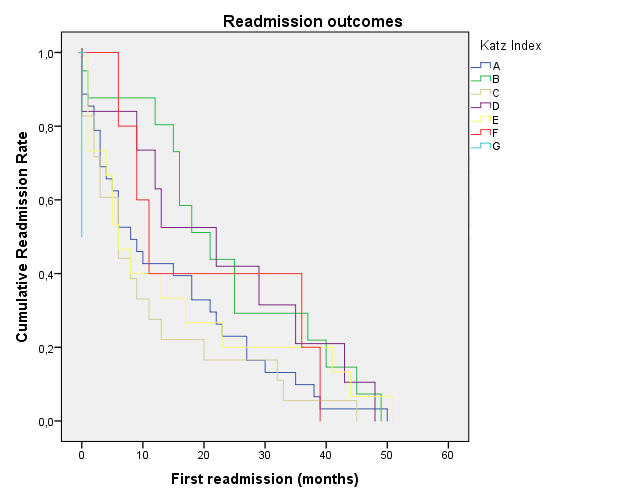


## Figure S1. Time until first readmission outcome on Katz index categories. P=0.033.
